# Unmet mental health needs in the general population: perspectives of Belgian health and social care professionals

**DOI:** 10.1186/s12939-020-01287-0

**Published:** 2020-09-29

**Authors:** Eva Rens, Geert Dom, Roy Remmen, Joris Michielsen, Kris Van den Broeck

**Affiliations:** 1 Collaborative Antwerp Psychiatric Research Institute (CAPRI), University of Antwerp, Antwerp, Belgium; 2 Department of Primary and Interdisciplinary Care (ELIZA), University of Antwerp, Antwerp, Belgium; 3 AZ KLINA, Brasschaat, Belgium

**Keywords:** Unmet needs, Mental health care, Primary care, Social work, Focus groups, Treatment gap, Barriers

## Abstract

**Background:**

An unmet mental health need exists when someone has a mental health problem but doesn’t receive formal care, or when the care received is insufficient or inadequate. Epidemiological research has identified both structural and attitudinal barriers to care which lead to unmet mental health needs, but reviewed literature has shown gaps in qualitative research on unmet mental health needs. This study aimed to explore unmet mental health needs in the general population from the perspective of professionals working with vulnerable groups.

**Methods:**

Four focus group discussions and two interviews with 34 participants were conducted from October 2019 to January 2020. Participants’ professional backgrounds encompassed social work, mental health care and primary care in one rural and one urban primary care zone in Antwerp, Belgium. A topic guide was used to prompt discussions about which groups have high unmet mental health needs and why. Transcripts were coded using thematic analysis.

**Results:**

Five themes emerged, which are subdivided in several subthemes: (1) socio-demographic determinants and disorder characteristics associated with unmet mental health needs; (2) demand-side barriers; (3) supply-side barriers; (4) consequences of unmet mental health needs; and (5) suggested improvements for meeting unmet mental health needs.

**Conclusions:**

Findings of epidemiological research were largely corroborated. Some additional groups with high unmet needs were identified. Professionals argued that they are often confronted with cases which are too complex for regular psychiatric care and highlighted the problem of care avoidance. Important system-level factors include waiting times of subsidized services and cost of non-subsidized services. Feelings of burden and powerlessness are common among professionals who are often confronted with unmet needs. Professionals discussed future directions for an equitable mental health care provision, which should be accessible and targeted at those in the greatest need. Further research is needed to include the patients’ perspective of unmet mental health needs.

## Background

Approximately one in four people experiences a mental disorder at some time during one’s life [1, 2]. However, not everyone who needs mental health care receives it. First, the need for mental health care has to be perceived [3–5]. Among those who recognize the need, a considerable amount seeks no professional help, and seeking help doesn’t guarantee appropriate care will be received [3–7]. The result of this cascade is a considerable care gap and unmet mental health needs (UMHNs) [7–13].

The majority of epidemiological studies in the literature investigating UMHNs used quantitative survey research. A first approach estimates ‘objective’ UMHNs and assesses the use of health care among people with a diagnosed mental health condition. For example, the authors of the European Study of Epidemiology of Mental Disorders (ESEMeD) use a low-threshold definition and describe an UMHN as the lack of use of any formal health care among individuals with a diagnosed mental health or substance use disorder which leads to considerable disability [10]. Following this definition, approximately 3% of the general population in high income countries have UMHNs [10, 12]. However, not all formal care may be sufficient or appropriate. Therefore, other studies have taken ‘minimally adequate treatment’ criteria into account, and found that more than half of the formal care received cannot be considered at least minimally adequate [14, 15]. This suggests that simply considering a need as being met when there has been any contact with formal care leads to an underestimation of the true level of UMHNs.

Another approach is to assess ‘subjective’ UMHNs by asking subjects whether they ‘felt a perceived need for mental health treatment in the past year that was not met’. This definition results in an estimated population level of UMHN of 5.6% [16]. Among those with a diagnosed mental health disorder, most needs are perceived as unmet, and this is especially the case for counseling needs such as psychotherapy [17–19].

Vulnerable groups are generally more likely to underuse or experience access barriers to health care, resulting in high levels of UMHNs [20]. Groups with a high level of UMHNs include the young and the elderly, ethnic or cultural minorities, people with poorer health and people with lower income [11, 21–23]. Also patients with more complex needs are less likely to receive appropriate care, such as those with a longer disease course, comorbidities and substance abuse [10, 11, 21, 22].

Previous studies identified several barriers that lead to UMHNs. These barriers can be broadly divided into two groups: attitudinal barriers and structural barriers [24]. Attitudinal barriers are demand-side barriers operating at the individual, household or community level and influence the demand for care [25, 26]. In contrast, structural barriers are supply-side barriers operating at the system-level and are beyond the individuals’ control [25, 26]. Attitudinal barriers, such as preferring to manage problems on one’s own, are mentioned more often as reasons for not seeking professional help than structural barriers [18, 24, 27, 28].

Not perceiving a mental health need is one of the most frequently cited reasons for not seeking professional help [27–30]. As can be expected, people with less disabling symptoms are least likely to seek help, but more worrying is that also a considerable amount of people with a severe mental health disorder do not perceive any need for mental health care [27, 28, 30].

The more severe the disorder, the more likely one is to face structural barriers to mental health care [27, 29]. Cost of services was the most often cited reason for UMHNs in a recent large-scale study in the United States [11]. In addition to increasing service provision, existing services therefore also have to be optimized in terms of access, use, effectiveness and efficiency [10].

Although it is clearly shown that UMHNs are widespread, qualitative explorations of UMHNs in the general population are sparse. Survey methods may hold the limitation that vulnerable groups, such as minorities or the poor, are hardest to reach for participation [31]. Underrepresentation of vulnerable groups could give rise to a biased prevalence of mental health needs and non-valid correlates of other measures [32]. Qualitative research with professionals working with vulnerable groups could identify correlates of UMHNs that are overlooked in epidemiological surveys.

This study therefore aims to explore UMHNs from the perspective of primary care, mental health care and social care professionals. Specifically, the study intends to identify which people with mental health problems do not receive appropriate or sufficient mental health care and why. Moreover, a qualitative approach may serve an improved understanding of quantitative findings. Professionals’ understanding of UMHNs and its determinants can ultimately support policy and strategies for optimally targeting those in the greatest need.

## Methods

Exploratory qualitative research was chosen to allow a broad investigation of UMHNs in the province of Antwerp, in the Flemish part of Belgium. Since 2018, new structures called primary care zones were developed, encompassing geographical entities covering approximately 100,000 inhabitants [33]. These regions are aimed at strengthening collaboration and coordination between local primary care professionals, organizations and secondary health care. UMHNs in one urban and one rural primary care zone were investigated using focus group discussions (FGDs) from October 2019 to January 2020.

A purposive sample of local primary care, mental health and social care professionals was recruited. Recruitment was done via the coordinators of the primary care zones and directly by e-mail by the researchers after an extensive online search. In addition, because no psychiatrist was able to attend the FGDs, psychiatrists were invited for an interview. Two heterogeneous FGDs took place in each primary care zone. Two semi-structured interviews took place with psychiatrists working in the urban primary care zone, as there was no available psychiatrist in the rural primary care zone. After the four FGDs and two interviews with a total of 34 participants, no new information emerged, and the researchers concluded saturation was reached.

The FGDs were conducted by two researchers (ER and KVdB) with a background in psychology. A semi-structured topic guide was used and consisted of simple, open questions prompting discussion about the following topics: groups with high unmet need (e.g., Which people are hardest to reach?), reasons for unmet need (e.g., Which barriers lead to unmet needs?), the quality and quantity of local care provision (e.g., Are there enough services in this area? How is the collaboration in this area?) and future directions (e.g., What should be done to meet those unmet needs?). FGDs lasted between 90 and 120 min each. Similar questions were asked in the interviews, which both took 60 min and were conducted by ER. All participants gave written informed consent and were offered a transfer of €50 afterwards.

All contacts were audio recorded and fully transcribed. All transcripts were thematically analyzed using NVivo. Transcripts were first fully inductively coded, i.e., without a pre-set codebook. Related codes were merged into categories. Subsequently, categories were grouped into meaningful themes. A deductive approach was used in this step, as some themes are partly based on the literature about different types of barriers in mental health care.

Credibility of the initial set of themes and subthemes was explored through discussion amongst the researchers and member-checking. Participants received a short report and were given the opportunity to (dis) agree and to share additional thoughts and remarks. Five participants responded to the invite to reflect on the initial theme set, of whom two agreed without remarks, and three gave some additional recommendations. This led to some small changes in the final theme set. The quotes in this paper were translated by ER. The study was approved by the medical ethics committee of Antwerp University Hospital (EC UZA 19/33/380).

## Results

### Participants

A total of 34 participants took part in the study, with a mean age of 43.5 years (*SD* = 11.1) and a mean of 14.0 years (*SD* = 8.1) of experience. Three categories of professionals participated: primary care workers (e.g., general practitioner, home health nurse, …), mental health workers (e.g., psychotherapist, psychiatrist, …) and social workers (e.g., children and youth services, community services, …). Sample characteristics are displayed in Table 1.

**Table 1:** Demographic characteristics of participants

	Urban			Rural		Total
	FGD1	FGD2	Interviews	FGD3	FGD4	
Setting						
Primary care	1	1	0	1	4	7
Mental health	4	0	2	1	1	8
Social work	4	7	0	3	5	19
Characteristics						
Age (mean)	47.0	42.9	32.0	47.2	41.4	43.5
Female/Male	7/2	7/1	1/1	5/0	9/1	29/5
Total number	9	8	2	5	10	34

### Findings

Five themes were identified: (1) socio-demographic determinants and disorder characteristics associated with UMHNs; (2) demand-side barriers associated with UMHNS; (3) supply-side barriers associated with UMHNs; (4) consequences of UMHNs; and (5) suggested improvements for meeting UMHNs.

#### Theme 1: socio-demographic determinants and disorder characteristics associated with UMHNs

UMHNs were considered overrepresented in some groups, due to several socio-demographic and disorder-related characteristics. A first major risk factor professionals mentioned was **poverty**. Non-reimbursed psychotherapy is too expensive, while waiting times in reimbursed services are long. Professionals state that poverty hinders help-seeking because mental health needs are subordinate to basic needs such as housing and food. Difficulties were mentioned distinguishing mental health needs from rather social needs, and the two are often intertwined.

*“I don’t think it’s about certain groups, but more … across all groups, when there is not enough financial capacity, I don’t think there is more necessity for this or that problem. Once people lack financial resources, it transcends all groups.” (psychotherapist, FGD1).*

Second, people with an **ethnic minority background** were seen as a hard-to-reach group for mental health care. Cultural differences in taboo and stigma and a lack of trust in professional care were identified as hindering factors for help-seeking in this group. People with a non-western background often present with indistinct physical complaints which are actually caused by underlying mental distress. Also language plays a major role. Several professionals expressed difficulties working with interpreters, and not speaking one of the national languages is often an exclusion criterion in mental health care. Professionals voiced concerns about severe trauma amongst the increasing number of refugees.

*“What we see in general practice is that people present with physical complaints that last very long because they have underlying psychological complaints about which they cannot talk because of cultural … also because of cultural differences, but also because no space is given to discuss those things, partly also because of language problems.” (general practitioner, FGD1).*

As regards disorder characteristics, professionals mentioned UMHNs were high in psychiatric patients with **complex care needs**. Patients with co-occurring mental or substance use disorders, or in whom a severe mental disorder is accompanied by problems in multiple domains, were found difficult to get into treatment. They do often not fit the right criteria, and some tend to be excluded due to their externalizing behavior. There is also a subgroup with complex needs who were pejoratively called ‘revolving door patients’ or ‘frequent flyers’ because they’re often re-admitted to psychiatric wards or crisis units.

*“We are often stuck with, uhm, revolving door patients as they say. Been admitted to all psychiatric institutions, not welcome anywhere anymore, drug problems on top. That is a particularly large group we can’t get away with.” (center for general wellbeing, FGD1).*

Also **long-term care needs** increase the risk of UMHNs, professionals argued. Some patients suffering from severe chronic mental disorders require long-term or even lifelong care. Such care needs are currently often unmet due to a capacity problem. Patient flow ceases due to limited outflow, causing saturated long-term care services. It was mentioned that long-term care can be low-intensive in stabilized conditions, as long as there is at least some follow-up of how a patient is living one’s life.

*“Trajectories that we start up today are not finished within two or three years, which also creates a waiting list. Because there are a lot of people with a request for long-term care, sometimes it’s just expressing one’s feelings like ‘today didn’t went well’.” (long-term care team, FGD3).*

Finally, professionals mentioned UMHNs are high in both young and old age. Waiting times in mental health care are a major problem in **children and youth** care. Generational problems are common, such that troublesome parenting situations lead to behavioral and emotional disorders in children. As regards youth, professionals noticed a gap in transition age. Protection of minors abruptly stops at the age of 18 and many vulnerable young people struggle with finding their place in society. Professionals in both primary care zones mentioned the phenomenon of young ‘couch surfers’ living in hidden homelessness by continuously sleeping at other people’s houses.

*“In particular those young people, those 18 to 25 year old’s who are left out everywhere and who actually need more care. They are excluded everywhere, they remain in special youth care or in foster care or disabled care, then they turn 18 and everything stops and there they are.” (children and youth services, FGD2).*

UMHNs are also high among the **elderly**. Professionals argued that it’s mainly a demand-side problem, as the oldest generation isn’t used yet to the idea of mental health care. Moreover, vulnerable elderly in nursing homes often lack appropriate mental health care. For example, one participant worked in a nursing home as a moral counsellor but actually dealt with complex psychiatric needs requiring specialized staff.

*“I can’t refer my people, to no one. My whole day is filled with conversations with people who are almost all tired of life. I’ve got people with Korsakov, with delusions, with psychoses, with everything you can imagine. And I am actually the help, and that’s where it stops. [ …*] *So the philosopher is the one who has to have a little chat with them to fix it.” (moral counsellor, FGD2).*

#### Theme 2: demand-side barriers associated with UMHNs

Receiving mental health care is often a matter of demanding care, but many people don’t actively seek help or prefer to deal with problems on their own. First, professionals argued that many people with mental health issues don’t **perceive a need for mental health care**. Insight into one’s needs is often a prerequisite for mental health care, especially for psychotherapy. Insight is in particular lacking in vulnerable groups with low mental health literacy. People often feel something is wrong but experience difficulties to put their concerns into words, or to formulate an explicit request for help.

*“And problem is also for those socially vulnerable: a screening is done or an intake, but those people should have a request for help, they have to be able to formulate it, where they are willing to work on, and that is so difficult for them.” (psychotherapist, FGD4).*

Some people with a mental disorder avoid or refuse any kind of professional help and are non-compliant to offered therapies. For these **care avoiders**, the situation may be worrisome and urgent enough for ‘interfering care’ to take place, i.e., care aimed at protecting them.

*“There is a long waiting list for the people who want to [be helped], and there’s an even longer waiting list for people who do not want to [be helped]. And the people who don’t want it, that’s often the people where it’s more urgent, where the problems are a lot more complex, where the most interfering care is needed because regular care won’t work.” (subsidized housing assistance, FGD2).*

Finally, **taboo and stigma** hinder people from disclosing mental health problems and seeking help. Although a slight positive evolution took place in recent years according to professionals, taboo remains a major barrier in some groups, such as people from non-western cultures and the elderly.

*“For us Belgians it’s still taboo, but in other countries it’s often even a much bigger taboo. You’re crazy, some people don’t want to talk with you anymore when you’re crazy, so then you actually can’t share it.” (family center, FGD2).*

#### Theme 3: supply-side barriers associated with UMHNs

Another theme emphasizes supply-side barriers in the health system that hinder access to adequate mental health care. First, **underfinancing of mental health care** resulting in the lack of structural resources was regularly mentioned as an important underlying factor for UMHNs. Yet, due to the creativity of health care providers, interesting local initiatives were installed, often financed by local organizations and authorities. This was particularly the case in the rural zone.

*“The offer of TEJO [*free therapy for youngsters, provided by volunteers]*, that’s fantastic. It works well and it reaches an enormous amount of young people, but at the same time it’s something to be bloody ashamed of as a society that it has to run on volunteers.” (children and youth services, FGD4).*

In addition to a lack of resources, professionals mentioned a **lack of time**. This is partly due to staff shortage and bureaucratic overload (administrative burden, sharp targets, the overload of rules and complex procedures). The situation resulted in high work pressure, overtime, and reduced quality of care.

*“So, we have to count like that: they have 36 hours of help on a yearly basis but making a report also counts, so that means about 3 hours per month. And this is how we, unfortunately, have to deal with it. And then I think, tailored care? There’s just no way to do it.” (children and youth services, FGD4).*

Moreover, professionals argued that a **fragmentated and suboptimally distributed mental health sector** contributes to the level of UMHNs in society. There are a variety of support initiatives, both in the public and private sector, but a comprehensive overview and coherence between the services are lacking. Ambiguity about the organizations’ responsibilities and offer adds to discontinuity of care, inappropriate referrals and false expectations. Moreover, concentration of services strongly differs from one region to another. Mobility impedes access to mental health care in rural areas which is mainly concentrated in the inner cities. Some organizations limit their services to inhabitants of a certain region.

*“You get the runaround, in this region there are none, there are no specialized centers except for some ambulatory care [ …] You have to go outside the region, and what do those regions tell us, and that makes some sense as well: ‘we first look within our own region to be able to follow up the aftercare better afterwards.’” (center for general wellbeing, FGD3).*

Also the **cost and limited reimbursement of primary mental health care** is an important factor according to professionals. In Belgium, primary mental health care is reimbursed for a maximum of eight sessions for light to mild psychological complaints. Many people, and in particular those in the highest need, are excluded in the system. On the other hand, cost is less of a barrier for psychiatric medication or for hospitalizations, because these services are reimbursed.

*“The first thing that perishes [when in debt] is actually the paid-for psychological care and so on, and then you notice very strongly in our trajectories that the fact that you can’t apply third party payment, that that’s simply the first criterium to cut something out.” (community work, FGD1).*

Subsidized services provide affordable mental health care but **waiting times are long,** ranging from months to even several years. Not providing care at the right time was thought to be an important factor adding to UMHNs. Professionals argued waiting lists are caused by insufficient capacity, but also result from suboptimal patient flows, including outflow problems.

*“Then they’re on the waiting list of the center of mental health care, which is currently two years around here, so that’s actually not a solution. Those people, when they are called, they can’t even remember why. [ …] The problem has further developed in the meantime or has landed somewhere else.” (regional coordination, FGD4).*

Another barrier is the use of **strict inclusion and exclusion criteria** in mental health care services. Facilities often have programs or wards focusing solely on delineated problems. This may however hinder access for people whose label is unclear or who do not fit into the right criteria. Professionals referred to a limited number of treatment places for people with dual diagnoses.

*“What I often notice in our target group is that we work a lot with people who fall somewhere in between. Those are very complex problems and a little bit of this and a little bit of that, and generational and so on. In the hospital, the crisis is often too heavy or not heavy enough, sometimes it’s too chronic or not chronic enough.” (children and youth services, FGD4).*

#### Theme 4: consequences of UMHNs

UMHNs lead to negative emotions in both patients and care providers, what can eventually make the situation even worse. **Crises** are often the result of an escalation of UMHNs. Professionals talked about ‘downward spirals’ caused by lack of care. Two extreme expressions of crises which were mentioned are involuntary commitments and suicides.

*“When involuntary commitments are used for which they were intended, then it’s a good system. But if there’s so much need and so much care and so much crisis that an involuntary commitment has to be used, then you will always be shutting the stable door after the horse has bolted.” (center for general wellbeing, FGD3).*

Participants often expressed **feelings of frustration and powerlessness** because of the large level of UMHN in society which they sometimes can do little about. They felt they have no impact on the length of waiting times, the access to mental health care for vulnerable groups and so on.

*“It’s also the powerlessness we feel as caregivers. I think GP’s frustrations are often about this, also my frustrations or anyone working with those difficult cases who can’t get in anywhere because of waiting lists, but also because they burned many bridges and caused trouble and that care providers tell them: ‘No, he can no longer come to us’.” (psychiatrist in center for mental health care, interview).*

Finally, all **involved services become overburdened**. Because of an excess demand of mental health care and limited access to specialized services, people with complex mental health needs often linger in primary care and social services. This is in particular a problem when the front-line is overloaded with people who fall through the cracks of the mental health care system but who actually need more than generalist care. Primary care then becomes not only the first, but also the last resort.

*“There is a full waiting list, we are backed into a corner. And this way it’s indeed having the conversations yourself, keeping contact, connecting, … But you know you’re not the right man at the right place, and I also lack knowledge. But not doing anything is no option at all. [ …] That’s very difficult, also because your team suffers a great deal from it.” (public social welfare center, FGD3).*

#### Theme 5: suggested improvements for meeting UMHNs

Several improvements were suggested for an optimal and more equitable mental health care. Some professionals argued that a redistribution of resources is needed in the Belgian mental health care sector. To begin with, a **redistribution between regions** should increase service provision in disadvantaged rural areas.

*“I notice that there are money flows to primary and secondary care and that there are nice initiatives, but that doesn’t count for all regions, and not for all target groups.” (psychotherapist, FGD1).*

Secondly, increasing resources for **low-threshold and outpatient mental health services** is needed to overcome the gap in accessible care. Mental health care was considered accessible when it’s affordable, when waiting time is limited and when referral is no prerequisite. As regards to affordability, extension of the reimbursement of psychotherapy was considered an important step.

*“I think that there is a big problem in that group of people you have to offer an accessible place to talk, which first was the intention of the centers of mental health care, to be the house in the street in which you can walk in to talk.” (psychiatrist in center for mental health care, interview).*

Professionals and patients also need a **comprehensive overview** of the available services, their target groups and organization type. Professionals suggested that a central referring instance could help, which is in contact with all regional services and has knowledge of criteria, procedures, etc.

*“What I think would be helpful is that we don’t have to call around to know where a patient can go, or who has an available bed, or ‘this is a difficult case and I don’t really know what to do with him’. [ …] Such a contact person who can guide us, because I lose a lot of time with it.” (general practitioner, FGD1).*

Professionals argued that more **outreaching care** is needed. Outreach in mental health care means that the care provider takes the initiative and reaches out to the vulnerable person instead of the other way around. Professionals mentioned a high need for outreach in worrisome care avoiders and ethnic minorities.

*“Street psychiatrists. People are registered from various organizations, public centers for social welfare or subsidized housing services report it and then psychiatry will ring those people’s doorbell without them having a care request themselves. [..] I think there can certainly still be made a movement, it’s on its way, but still.” (psychiatrist in hospital, interview).*

We should invest in **multidisciplinary and intersectoral collaboration and coordination**. People with severe mental disorders often need support in multiple life domains, but it became clear that collaboration is currently not optimal. Professionals mentioned a need for case management, in particular in complex cases or people with a social vulnerability.

*“That’s why I think multidisciplinary teams are so important. You can make sure his psychotic complaints are under control with pills, but you have not treated someone that way, I think. You have to socially support, that he has a network, [ …] hopefully a job but otherwise daily activities, that his house stays in order a bit and the bills paid and …*” *(psychiatrist in hospital, interview).*

Related to multidisciplinarity is the importance of **continuity of care**, which refers to how care is connected over time. Aftercare should be optimized, especially after a hospital stay. It was argued it benefits the patient if one can rely on the same services and caregivers over time.

*“I think a great need is continuity of care. [ …] Everyone tries short-term, finish as quick as possible, mostly little continuity in care. [ …*]. *People feel safer if there’s still a door ajar somewhere.” (psychotherapist, FGD4).*

A need for **tailored care** was mentioned as well, providing adequate care at the right time in the right context, thereby following stepped care principles. It was mentioned that currently some procedures or rules hinder quick and flexible care, such as a fixed number of sessions per client.

*“Intensive when it’s not going well, and when people say they feel secure about themselves again, okay, the frequency simply goes down again and we’ll see what’s needed. And then you also empower people.” (children and youth care, FGD4).*

Increased attention is needed for **cultural-sensitive care**, for example during professional’s education. Moreover, the use of interpreters in mental health care should be better supported.

*“You have to know what a djinn is. It’s about, how can you connect with people in your neighborhood, with the audience you work with? I guarantee, when I would do quality research [about cultural sensitivity], that we would be appalled.” (center for general wellbeing, FGD1).*

Professionals argued that **a strong focus on prevention** will benefit the mental health of the population in the long run. Today, the emphasis is largely on curative care. It was argued that preventive interventions are a hard sell because its impact is less visible and measurable.

*“I think a lot is still possible in terms of prevention, because often when something happens you immediately have a crisis or situations that are suddenly very urgent. And we notice that by making it possible to talk about psychological complaints in the form of recognizable symptoms, that it also lowers the barrier to seek help.” (Agency for Integration and Civic Integration, FGD4).*

Finally, the importance of **recovery-oriented care and informal supportive networks** for people with mental health problems was stressed. Good practices such as buddy systems, support groups and investing in neighborhood cohesion were mentioned.

*“I am thinking of working more with people’s network and people’s own strength. [ …] Because assistance is not forever, right. When people have significant others, professional or not professional, you notice they are also helped in the long term.” (subsidized housing assistance, FGD2).*

## Discussion

In this study, Belgian professionals with a background in social work, mental health and primary care discussed UMHNs in the general population through four FGDs and two interviews. The findings should be interpreted in the context of the following limitations. First, the participants were predominantly female, and it’s unclear whether and how this influenced the findings. Second, the sample did only include care providers and the findings are therefore not confirmed from a care perceiver perspective. We believe professionals have a good idea of the unmet needs and barriers in their primary care zone, but their perspectives still contain assumptions about their patients’ and clients’ motives. This is especially true for attitudinal barriers people with mental health problems are confronted with, as these barriers are less visible to professionals. Future research will examine whether patients’ perspectives correspond to those of professionals.

At the same time, the variety of professional backgrounds and settings of the professionals is a strength of the study. The inclusion of non-mental health professionals stems from the idea that many vulnerable people with mental health problems don’t reach mental health care services, but remain in social and primary care services.

It’s important to take the rather complex Belgian context into account. The governance of mental health is fragmentated over federal and regional authorities. Health care is generally financially accessible as health insurance is compulsory, but large out-of-pocket payment and limited coverage of certain ambulatory mental health care services remain a concern in Belgium. The findings should therefore not be generalized to other countries.

Finally, analyses were performed by two psychologists (ER and KVdB). However, member checking and discussion of the themes with a psychiatrist (GD), a sociologist (JM), and a general practitioner (RR) add to the study’s credibility.

Despite these limitations and contextual remarks, we can conclude that our findings are in line with those of epidemiological studies. According to our participants, UMHNs are most prevalent in vulnerable groups such as people living in poverty, ethnic minorities, and in the young and old age groups [11, 21, 22]. These are groups for which multiple barriers are often present, both on the supply- and demand-sides. For example, professionals argued ethnic minorities are often hindered by attitudinal factors such as taboo and stigma, but system-level barriers such as language also hinder access. A specific group with high UMHNs which was mentioned in both primary care zones, but which is not mentioned in the literature, is the group of young sofa surfers. Interestingly, a recent study in Flanders confirms that homelessness is often hidden, especially in rural areas and among young people [34].

Regarding disorder characteristics, those with more complex or chronic psychiatric needs were mentioned as groups with high UMHNs, and this was mainly attributed to structural factors such as strict criteria and capacity of long-term care facilities. This is in line with previous findings reporting that structural barriers dominated for severe cases, but not for mild or moderate cases [27] Professionals working with people with co-occurring substance abuse disorders indicated that it’s difficult to find appropriate care for this group, especially as mental health treatment is often refused when substance abuse is present. This access barrier may partly explain why approximately half of the people with a co-occurring mental health and substance use disorder receive neither mental health care nor substance abuse treatment [13].

Our study confirms that attitudinal barriers and not recognizing a need for care play a major role in the development of UMHNs. Interventions focusing on mental health literacy and de-stigmatization of mental health problems may help to recognize needs and to overcome attitudinal barriers [6, 11, 35]. ‘Worrisome care avoiders’ were particularly mentioned in this context as a group with high UMHNs for which specialized outreach interventions are needed. Related to this finding, a review of ‘difficult patients’ in mental health care identified ‘unwilling care avoiders’ as one of the three difficult groups and suggests psychotic disorders are common among this group [36].

Several types of structural barriers were identified, both on a higher health system-level (e.g., distribution of services) and on a lower organizational level (e.g., strict criteria). Cost of non-subsidized services and waiting times of subsidized services were mentioned as two important factors impeding access to care. It was argued that vulnerable groups are disproportionally affected by structural barriers and that this introduces inequities in mental health care. For example, people with insufficient financial resources are unable to bridge long waiting lists by consulting non-reimbursed psychologists.

An important finding is that feelings of frustration and powerlessness are common among professionals. The workload is high in all involved settings, and an increasing demand for efficiency within organizations means that professionals sometimes have to make decisions that go against their ideals. This relates to the phenomenon of ‘moral distress’, which occurs when health providers cannot carry out what they believe to be the right thing to do [37]. Moreover, some vulnerable people with complex mental health needs circulate in the social work or primary care circuit without being able to access specialized mental health care. As a result, non-mental health professionals felt as if they have to go beyond their core responsibilities to meet their client’s mental health needs. This phenomenon of ‘last-line care’ for the most vulnerable needs further investigation.

Professionals were asked what should be changed in order to meet the needs that are currently unmet. Professionals made several recommendations for the broader mental health system. Most of the responsibility is borne by the top of the hierarchy, such as governmental decisions on the reimbursement of psychotherapy and the allocation of resources among regions and sectors.

Services were considered accessible by professionals when the cost is low, no referral is needed and when one can be helped quickly. However, access can also be interpreted more broadly, with the inclusion of demand-side factors. For example, Levesque et al. define access from a multi-level perspective as “the opportunity to identify health care needs, to seek health care services, to reach, to obtain or use health care services and to actually have the need for services fulfilled” [38]. Little is known about interactions between barriers, but there is a possibility that changes in supply-side barriers are able to modify demand-side barriers. This idea requires further research.

Furthermore, professionals stressed the need for multidisciplinary teams and intersectoral collaboration. Some patients receive care from multiple sources that are often not coordinated, which can be confusing for both the patient and the care providers. Mental health care should also be tailored to a patient’s needs, but professionals admitted that in practice they are too often bound by non-flexible procedures and trajectories. Professionals believe that currently far too little is done about prevention, partly because the need for curative care is high and budgets and time are limited. Also needs for continuity of care, cultural sensitivity of care and recovery-oriented care were stressed. For those who are hardest to reach or avoid mental health care, professionals believe more outreaching and in some cases even interfering care is needed.

Perspectives of UMHNs are similar in the rural and urban primary care zone, although there is a greater focus on the regional distribution of services and a lack of resources in the rural zone. Professionals in the rural primary care zone argue that the area is historically disadvantaged compared to others. Creative collaborations were created to meet this shortage.

As part of the Belgian mental health reform, several of the recommendations have already been put to practice in the form of mobile teams. Mobile teams are based on the Assertive Community Treatment (ACT) model and are multidisciplinary teams that either provide outreaching recovery-oriented care in the home environment for people with long-term severe mental health conditions who are often difficult to reach, or short-term crisis care for people with acute psychiatric problems [39, 40]. Although mobile teams are still developing in Belgium, it was cited as a good and promising practice if its capacity is further increased.

Overall, our findings add to a better understanding of the prevalence and determinants UMHNs in Belgium from the perspectives of professionals. Insight into UMHNs in the general population is highly relevant for realizing an equitable access to mental health care, in which all mental health needs are timely recognized and cared for. Future qualitative studies should include people with mental health problems, and especially those who have difficulty accessing mental health care services. Triangulation of the care provider perspective with the care receiver perspective will add to credibility and give a more balanced picture of the situation.

## Data Availability

Not applicable.

## Abbreviations

UMHN: Unmet mental health need
FGD: Focus group discussion
